# Flexible Textile-Based Organic Transistors Using Graphene/Ag Nanoparticle Electrode

**DOI:** 10.3390/nano6080147

**Published:** 2016-08-16

**Authors:** Youn Kim, Yeon Ju Kwon, Kang Eun Lee, Youngseok Oh, Moon-Kwang Um, Dong Gi Seong, Jea Uk Lee

**Affiliations:** 1 C-Industry Incubation Research Center, Korea Research Institute of Chemical Technology (KRICT), Daejeon 34114, Korea; younkim@krict.re.kr (Y.K.); kyj0905@krict.re.kr (Y.J.K.); 2 Composites Research Division, Korea Institute of Materials Science (KIMS), Changwon 51508, Korea; leeke88@kims.re.kr (K.E.L.); youngsoh@kims.re.kr (Y.O.); umk1693@kims.re.kr (M.-K.U.)

**Keywords:** e-textile, graphene oxide, textile composite, textile transistor

## Abstract

Highly flexible and electrically-conductive multifunctional textiles are desirable for use in wearable electronic applications. In this study, we fabricated multifunctional textile composites by vacuum filtration and wet-transfer of graphene oxide films on a flexible polyethylene terephthalate (PET) textile in association with embedding Ag nanoparticles (AgNPs) to improve the electrical conductivity. A flexible organic transistor can be developed by direct transfer of a dielectric/semiconducting double layer on the graphene/AgNP textile composite, where the textile composite was used as both flexible substrate and conductive gate electrode. The thermal treatment of a textile-based transistor enhanced the electrical performance (mobility = 7.2 cm^2^·V^−1^·s^−1^, on/off current ratio = 4 × 10^5^, and threshold voltage = −1.1 V) due to the improvement of interfacial properties between the conductive textile electrode and the ion-gel dielectric layer. Furthermore, the textile transistors exhibited highly stable device performance under extended bending conditions (with a bending radius down to 3 mm and repeated tests over 1000 cycles). We believe that our simple methods for the fabrication of graphene/AgNP textile composite for use in textile-type transistors can potentially be applied to the development of flexible large-area electronic clothes.

## 1. Introduction

Electrical textile (e-textile) has been considered as an ideal platform for flexible and wearable electronic devices on account of its light weight, flexibility, and comfort [1]. To date, many research groups have shown the feasibility of e-textile structures in various applications in organic field-effect transistors (OFETs) [2], organic photovoltaics (OPVs) [3], rechargeable batteries [4], and organic light-emitting diodes (OLEDs) [5]. From these studies, it has been determined that developing electronic materials with textile form and a multilayered assembly are key requirements for the realization of e-textile-based clothes.

In previous reports [6], electrolyte-gated textile transistors were demonstrated by using Au microwire as gate electrode. However, these devices showed poor flexibility and limitation for mass production, owing to the use of expensive Au wire electrode and complex micro-fabrication processes. Very recently, Oh et al. reported the fabrication of highly flexible photosensors on a commercially available textile substrate using photoresponsive semiconducting polymer nanofibers as the photoactive layers [7]. They also fabricated large-area photosensor arrays on textile substrate, which could be very practical for use as wearable photo imaging or scanning devices. However, for the construction of devices on the textile substrate, additional procedures are required, including the flattening of polyethylene terephthalate (PET) textile with a poly(dimethylsiloxane) (PDMS) layer and gate electrode deposition by thermal evaporation on buffered PET textile. To make the textile devices more practical, a facile fabrication process for a multifunctional textile composite should be developed.

In this work, we report on a quite simple method of fabricating highly conductive and flexible textile composites for the realization of wearable textile-based OFETs. The textile composites prepared by vacuum filtration and wet-transfer of graphene oxide (GO) on flexible PET textile, in association with embedding Ag nanoparticles (AgNPs), exhibited very low electrical resistance (4 Ω/γ) and outstanding flexibility (only 7.5% increase of resistance after 1000 cycles of bending test). A flexible organic transistor can be developed by direct transfer of a dielectric/semiconducting double layer on the graphene/AgNP textile composite, where the textile composite was used as both flexible substrate and conductive gate electrode. The textile-based transistor showed high electrical performance (mobility = 7.2 cm^2^·V^−1^·s^−1^, on/off current ratio = 4 × 10^5^, and threshold voltage = −1.1 V) and stable device performance after 1000 cycles of bending. We believe that our simple methods for the fabrication of graphene/AgNP textile composite for use in textile-type transistors can potentially be applied to the development of flexible large-area electronic clothes.

## 2. Results

### 2.1. Prepratation of Graphene/Ag Nanoparticle Electrode on PET Textile

**Figure 1:**
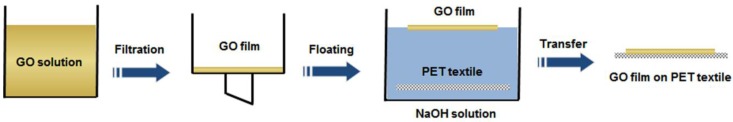
Schematic illustration of the vacuum filtration and subsequent transfer of graphene oxide (GO) film on a flexible textile substrate.

Figure 1 shows the overall process for the fabrication of graphene films on a flexible textile substrate using vacuum filtration and direct transfer methods. The vacuum filtration method is one of the most efficient and convenient techniques to fabricate thin graphene oxide films. GO films were transferred onto the mono-filament PET textiles, according to the method reported by Jung’s group [8]. Commercially available PET textile (filament diameter ~40 µm, pore size ~60 µm) was utilized because of its high flexibility (see Supplementary Information; Figure S1).

**Figure 2:**
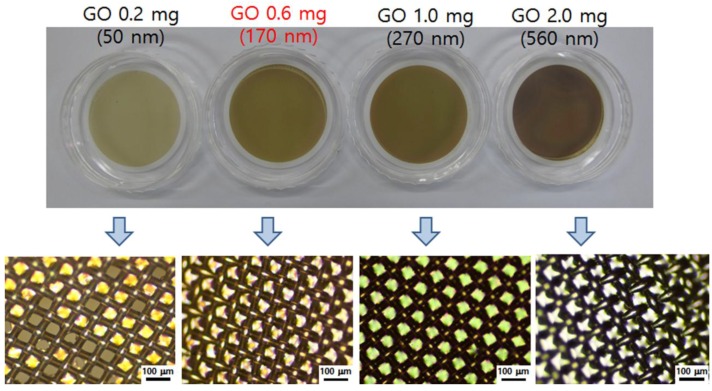
Photographs of GO films on anodic aluminum oxide (AAO) membranes with different amounts of GO (**top**). Optical microscope images of GO films on polyethylene terephthalate (PET) textiles (**bottom**).

Figure 2 shows the GO films on anodic aluminum oxide (AAO) membranes and PET textiles. To prepare the transparent and stable GO film, various GO solutions were vacuum-filtered through the AAO membrane by varying the concentration of GO solutions. We used an aqueous solution of NaOH in order to safely transfer the GO films to the PET textiles, since the NaOH solution not only removes the AAO membrane filter but also assists the fusion of GO films onto the PET textiles by slightly dissolving the bottom layer of GO films (see Supplementary Information; Figure S2). When the GO film was too thin (GO 0.2 mg, thickness ~50 nm), we observed that the GO film was easily torn down during the transfer process and could not cover the pores of the PET textile completely, whereas the thick GO film (GO 2.0 mg, thickness ~560 nm) could not merge with the PET textile because of the lack of flexibility. The thin film with 0.6 mg of GO (thickness ~170 nm) showed good coverage, flexibility, and transparency on the PET textile.

**Figure 3:**
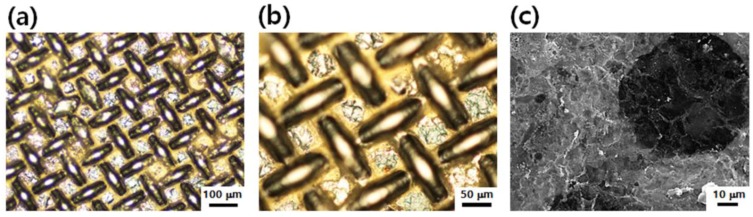
(**a**,**b**) Optical microscopy (OM) images and (**c**) scanning electron microscopy (SEM) image of graphene/AgNP textile composite.

To improve the electrical conductivities of the GO films on PET textiles for flexible electronic applications, the GO films (0.6 mg) were chemically reduced, and then embedded with silver nanoparticles (AgNPs). By introducing a silver precursor (AgCF_3_COO in ethanol) to the reduced graphene oxide (rGO) films and then converting into AgNPs following previously reported methods [9], we could develop graphene/AgNP composite films in which the AgNPs embedded onto both the outer surface and the inside of the graphene films. Figure 3 exhibits the composite film composed of rGO and AgNPs on PET textile (graphene/AgNP textile composite). The rGO/AgNP composite film coating on the PET textile maintained well without any distinguishable damages after chemical reduction and embedding of AgNPs. Furthermore, when we examined the microstructure of graphene/AgNP textile composite by scanning electron microscopy (SEM, Figure 3c), we could observe that the pores of the PET textile were completely covered by composite film. Although an excess amount of AgNPs was not observed on the graphene film due to the mild reduction reaction of AgNPs, the mean values of electrical resistance of the composite film were dramatically reduced from 5 kΩ/sq to 4 Ω/sq after embedding the AgNPs. Through the simple transfer of GO film and the subsequent embedding of AgNPs, we could prepare the highly conductive graphene/AgNP electrode on PET textile.

Since high flexibility is one of the important requirements for wearable devices, we investigated the effect of bending on the electrical properties of graphene/AgNP textile composites. Figure S3 (see Supplementary Information) displays the change of the electrical resistance of the textile composites as the number of bending cycles (bending radius = 3 mm) increased. The electrical resistance of the textile composites showed a small increase after 20 cycles of bending test and then maintained the stable value of 4.3 Ω/sq. The high electrical conductivity and durability to the repeated bending was attributed to the highly porous structures of rGO films, which provide room to incorporate enough AgNPs inside of the graphene layers.

### 2.2. Prepratation of Transistor Devices Using Graphene/AgNP Textile Composites

**Figure 4:**
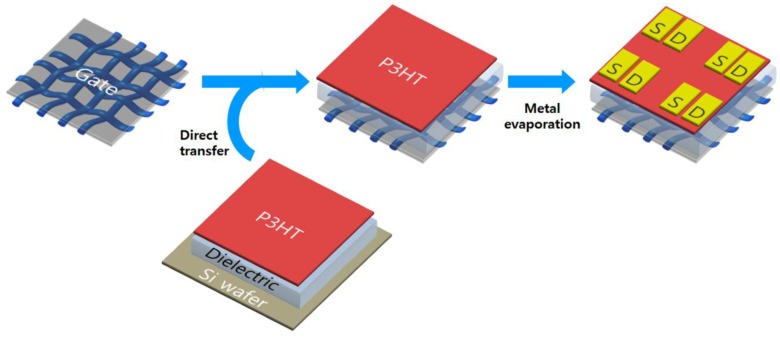
Schematic illustration of the fabrication process of the transistor device based on the graphene/silver nanoparticle (AgNP) textile composites. P3HT: Poly(3-hexylthiophene); S: Source electrode; D: Drain electrode.

In order to utilize the outstanding electrical conductivity and flexibility of the graphene/AgNP textile composite, we fabricated an organic transistor by using the textile composite as both gate electrode and flexible substrate. Transistor devices were fabricated with a bottom-gate, top-contact geometry with the composite film gate electrode, as schematically depicted in Figure 4. Poly(3-hexylthiophene) (P3HT) was chosen as the solution-processable *p*-channel semiconductor [10]. We also used an elastic ion gel layer based on poly(vinylidene fluoride-*co*-hexafluoropropylene) P(VDF-HFP) and the ionic liquid 1-ethyl-3-methylimidazolium bis(trifluoromethylsulfonyl)amide ([EMI][TFSA]) [11] as both high capacitance gate dielectric layer and mechanically robust transporter for the channel material. After the successive spin-coating of the ion gel and the P3HT layers onto a washed silicon wafer, the double layer was cut with a razor blade and transferred onto the graphene/AgNP electrode on PET textile for direct contact between the dielectric layer and the textile electrode [12]. This procedure provided a convenient and solvent-free route to simultaneously incorporate the active channel and the ion gel layers in the transistors without any contamination of each component. The fabrication of textile-based transistors was completed by simply patterning the Au source and drain electrodes (thickness ~40 nm) through a shadow mask by thermal evaporation onto the P3HT layer.

**Figure 5:**
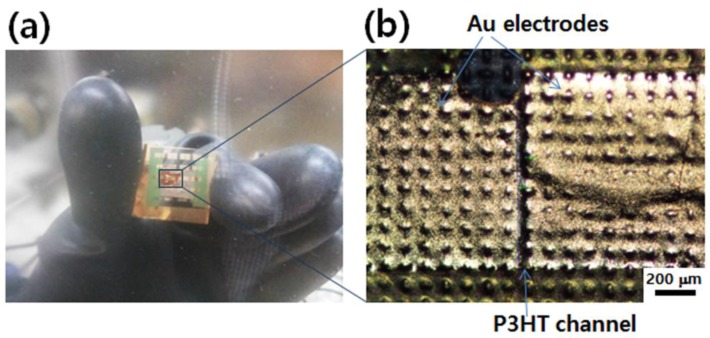
Photograph (**a**) and optical microscopy image (**b**) of textile-based transistor device.

Figure 5 displays a photograph and optical microscopy images of the transistor device developed in this study. For an accurate measurement of the electrical characteristics of the textile-based transistors, the device was fixed on the glass slide using 3M tape. The thickness of the P3HT and ion gel layer was 60 nm and 11 µm, respectively. The channel length (*L*) and width (*W*) was 50–100 µm and 800–1000 µm, respectively. The projection of the weaving morphology of the PET textile after the gold electrode deposition confirms that every layer of the device was compactly assembled by the simple transfer and deposition procedures.

**Figure 6:**
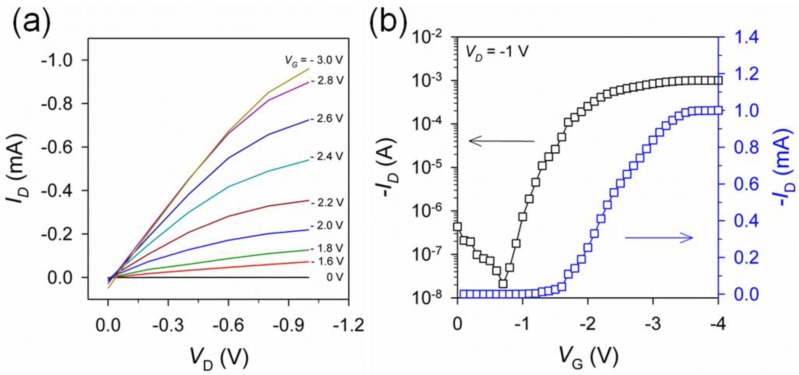
(**a**) *I*_D_-*V*_D_ and (**b**) *I*_D_-*V*_G_ characteristics of the textile-based transistor device.

Figure 6 shows the typical output (*I*_D_-*V*_D,_ where *I*_D_ is the drain current and *V*_D_ is the drain voltage) and transfer (*I*_D_-*V*_G,_ where *V*_G_ is the drain voltage) characteristics of the textile-based transistors. The well-defined gate modulation in the output curves (Figure 6a) reveals the ohmic contact between the composite film electrode and the transferred semiconductor/dielectric layers. The transistor device exhibited a saturation current of 1.0 mA at *V*_G_ = −3 V and *V*_D_ = −1 V. To evaluate the electrical characteristics of the transistors, such as the charge carrier mobility (μ_h_), on/off current ratio (*I*_on_/*I*_off_), and threshold-voltage (*V*_T_), the drain current was measured while sweeping *V*_G_ from 0 V to −4 V at a rate of 33 mV·s^−1^ and a constant *V*_D_ value of −1 V (Figure 6b). Despite the manual fabrication process in ambient conditions, the transistors made of the composite film electrode showed a reasonably high *I*_on_/*I*_off_ of 4.5 × 10^4^ and low *V*_T_ values around −1.5 V. From the slope of the *V*_G_ vs. |*I*_D_| curves obtained for more than five devices, the average field-effect mobility was calculated to be 2.7 cm^2^·V^−1^·s^−1^, which is much higher than those reported in other P3HT-based transistors gated with conventional dielectrics (0.1–0.01 cm^2^·V^−1^·s^−1^) [13], but are comparable to other recent results on electrolyte-gated polymer transistors [14]. It has been speculated that the high mobility value in this result is due to the penetration of ions from the ion gel dielectric into the active channel that fills the carrier traps and acts as a dopant in the P3HT film [15,16].

**Figure 7:**
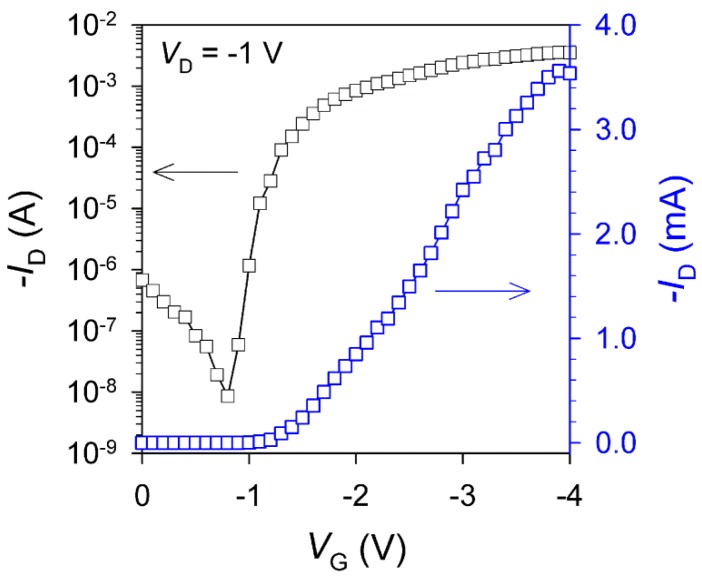
*I*_D_-*V*_G_ characteristics of the textile-based transistor device after thermal annealing at 80 °C for 15 min.

One approach toward improving the performances of the organic electronic devices (e.g, organic thin film transistors, organic light emitting diode [17], and organic photovoltaics [18]) is postproduction heat treatment. In order to enhance the device performance of the textile-based transistors, we thermally annealed the device at 80 °C for 15 min, which is a higher temperature than the glass transition temperature (*T*_g_) of P3HT (*T*_g_ = 12 °C) and PVDF (*T*_g_ = −115 °C) polymer layers [19,20]. From the optical microscopy image of the textile-based transistor device after thermal annealing (see Supplementary Information; Figure S4), clearer weaving morphology of the PET textile was observed over the gold electrodes, since the stronger interfacial adhesion between the component layers was developed by the thermal annealing. Furthermore, *V*_T_ was decreased to −1.1 V after thermal annealing, and the decrease of off-current and increase of on-current resulted in the enhancement of on/off current ratio and hole mobility of 4 × 10^5^ and 7.2 cm^2^·V^−1^·s^−1^, respectively (Figure 7). The improvement of device performance was ascribed to the crystallization and molecular orientation of the semiconducting polymer and the decrease of the contact resistance between the component layers induced by the thermal annealing. These results verify that the textile-based devices made by simple transfer method are applicable to the field-effect transistor (FET) device. Table 1 lists the detailed electrical properties of the textile-based transistors according to the thermal annealing.

**Figure 8:**
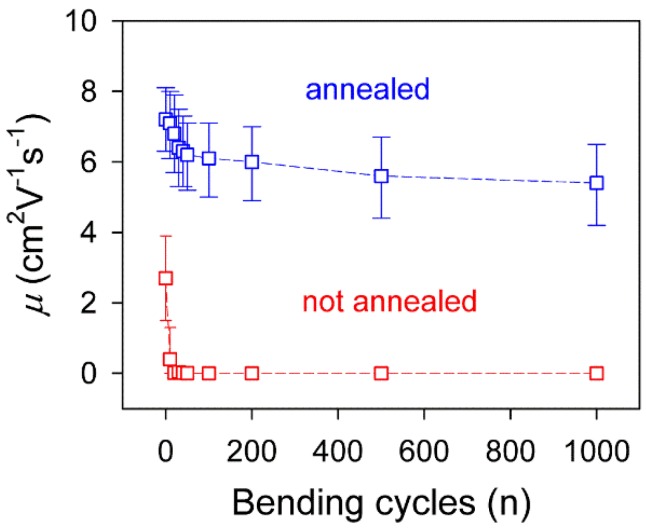
Changes in the hole mobilities of the thermally-annealed transistor (blue square) and not-annealed transistor (red square) depending on the bending cycle.

In addition, we measured the changes of device performance of the textile-based transistor after a cyclic bending test, because flexibility is critical for practical applications (Figure 8). In order to develop organic electronic devices with high flexibility, all of the components should be flexible and mechanically stable with intimate interfacial adhesion. The transistors without thermal treatment showed very unstable device performance; a considerable decrease of hole mobility was observed after bending the device to a radius of 3 mm for 10 cycles, which was because the upper layer (ion gel, P3HT, and Au electrodes) was peeled off from the graphene/AgNP textile composites. In contrast, the device performance of the annealed textile-based transistors maintained up to 70% of the original values through 1000 cycles of bending. From the bending radius of 3 mm and device thickness (90 µm), it is calculated that the annealed device underwent a strain of 0.014 repeatedly during the bending test. The outstanding durability of the annealed textile-based transistors originates from the good mechanical flexibility of every component and the closely-packed device structure of the textile-based transistor.

## 3. Materials and Methods

**Materials.** All materials were purchased from Sigma-Aldrich (St. Louis, MO, USA), except for the GO aqueous solution, which was purchased from Angstron Materials Inc. (Dayton, OH, USA). AAO membrane (200 nm pore size, 47 mm diameter; Whatman (Buckinghamshire, UK), PET textile (Textoma, Inc., Daegu, Korea), and Poly(3-hexylthiophene) (M_W_ = 50 K, Rieke Metals Inc., Lincoln, NE, USA).

**GO film transfer on PET textile.** GO films on PET textiles were prepared via vacuum filtration of aqueous GO solution and direct transfer onto PET textile, using the previously reported method [8]. Briefly, GO (0.2, 0.6, 1.0, 2.0 mg) in aqueous solution was first vacuum-filtered through an AAO membrane. The dried GO film was placed into a bath of 3 M NaOH. The AAO membrane was then dissolved, and the thin GO film was floated on the surface of the NaOH solution. The NaOH solution was exchanged with deionized water by recirculation until the pH was near 7.0. A piece of the PET textile (4 × 4 cm^2^) was immersed into the water and allowed to sink to the bottom of bath. As the water was drained, the floating GO film slowly descended and attached onto the PET textile. The GO film-PET textile was dried in ambient conditions.

**Graphene/AgNP composite film preparation on PET textile.** The dried GO films on PET textile were chemically reduced by exposing the films to hydrazine vapor (100%) at 90 °C for 2 h. To fabricate graphene/AgNP composite films, the rGO films on PET textile were dipped into a AgCF_3_COO solution in ethanol (15 wt %) for 30 min. After 5 min of drying in air, the films were exposed to hydrazine vapor (100%) at 90 °C for 2 h to reduce the adsorbed Ag ions to AgNPs.

**Fabrication of textile-based transistor.** Silicon wafer was cleaned by piranha solution to remove any organic contamination and treated with oxygen plasma to introduce a hydrophilic surface. The ion gel layer was prepared by first codissolving P(VDF-HFP) and the ionic liquid, [EMI][TFSA] in acetone (the weight ratio between the polymer–ionic liquid–solvent was kept at 1:4:7), and then spin-coated on the washed Si wafer at 1000 rpm for 1 min. Spin-coated ion gel layers were placed in a vacuum at 70 °C for 24 h to remove the residual solvent. Regioregular P3HT was spin-coated at 2000 rpm for 60 s from chloroform solution (10 mg·mL^−1^) on ion gel layer. The double layer of ion gel and P3HT was cut with a razor blade, and then transferred onto the graphene/AgNP textile composites for direct contact between dielectric layer and the composite film electrode. To complete the fabrication of textile-based transistor devices, Au source and drain electrodes with a thickness of 40 nm were thermally deposited onto the P3HT through a shadow mask. The devices were then annealed at 80 °C for 15 min on a digital hot plate under nitrogen atmosphere inside a glove box.

**Characterization.** Optical microscope observations were performed with a Nikon ECLIPSE LV150N (Tokyo, Japan). SEM images were taken on a JEOL JSM5800 (Tokyo, Japan). The electrical properties of graphene/AgNP composite films were characterized using a four-point probe measurement system (Napson, CRESBOX, Tokyo, Japan). The bending test was carried out with a home-made two-point bending device and a high-precision mechanical system. The measurement of the current–voltage characteristics of the textile-based transistor devices were carried out at room temperature in a N^2^-atmosphere glove box using a MST-5500B probe station (Gyeonggi-Do, Korea) and Keithley 4200-SCS (Solon, OH, USA). From the slope of the *V*_G_ vs. |*I*_D_| curves obtained for more than five devices, the average field-effect mobility was estimated in the linear regime (*V*_D_ = −1 V) from the following equation [21]:

(1) $$I_{D}=\mu \frac{W}{L}C_{i}V_{D}\left(V_{G}-V_{T}\right)$$

where *I*_D_ is the drain current, μ is the field-effect mobility, *C*_i_ (9 µF·cm^−2^) is the specific capacitance of the ion gel dielectric film, *V*_D_ is the drain voltage, *V*_G_ is the gate voltage, *V*_T_ is the threshold voltage, and *W* and *L* are the channel width and length, respectively.

## 4. Conclusions

In summary, we fabricated multifunctional textile composites by vacuum filtration and wet-transfer of graphene oxide on flexible polyethylene terephthalate textile in association with embedding of Ag nanoparticles, which showed high electrical conductivity and outstanding flexibility. A flexible organic transistor can be developed by direct transfer of a dielectric/semiconducting double layer on the graphene/AgNP textile composite, where the textile composite was used as both flexible substrate and conductive gate electrode. The thermal treatment of the textile-based transistor enhanced the electrical performance and bending durability due to the improvement of interfacial properties between conductive textile electrode and ion-gel dielectric layer. We believe that our simple methods for the fabrication of graphene/AgNP textile composite for use in textile-type transistors can potentially be applied to the development of flexible large-area electronic clothes.

## Figures and Tables

**Table 1:** Average hole-mobilities, on/off current ratios, and threshold voltages for textile-based transistors according to thermal annealing.

Treatment	μ_h_ (cm^2^·V^−1^·s^−1^)	*I*_on_*/I*_off_	*V*_T_ (V)
Without annealing	2.7	4.5 × 10^4^	−1.5
With annealing	7.2	4 × 10^5^	−1.1

## Supplementary Materials

The following are available online at http://www.mdpi.com/2079-4991/6/8/147/s1.
