# Supplementary material for: Flexible Textile-Based Organic Transistors Using Graphene/Ag Nanoparticle Electrode
Source: Nanomaterials (Basel). 2016 Aug 16;6(8):147. doi: 10.3390/nano6080147 (PMC5224629; doi:10.3390/nano6080147)
Supplement: Supplementary file 1 [file nanomaterials-06-00147-s001.pdf]

Supplementary Materials: Flexible Textile-Based Organic Transistors Using Graphene/Ag Nanoparticle Electrode

Youn Kim, Yeon Ju Kwon, Kang Eun Lee, Youngseok Oh, Moon-Kwang Um, Dong Gi Seong and Jea Uk Lee

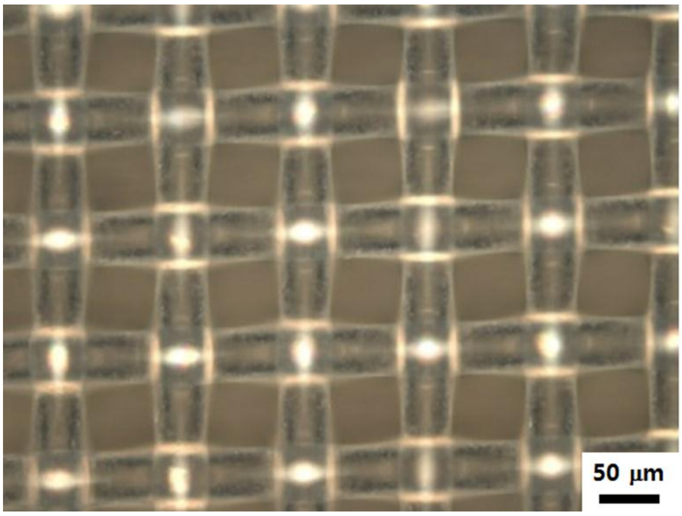

Figure S1. Optical microscopy (OM) image of monofilament polyethylene terephthalate (PET) textile.

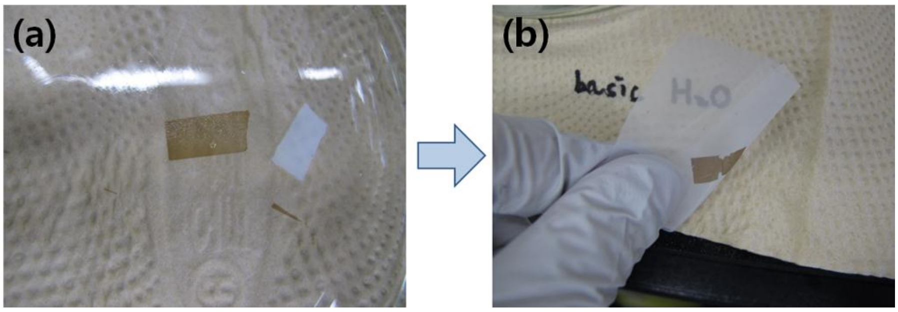

Figure S2. (**a**) Photographs of (**a**) floated graphene oxide (GO) film on NaOH solution; and (**b**) GO film completely combined with PET textile.

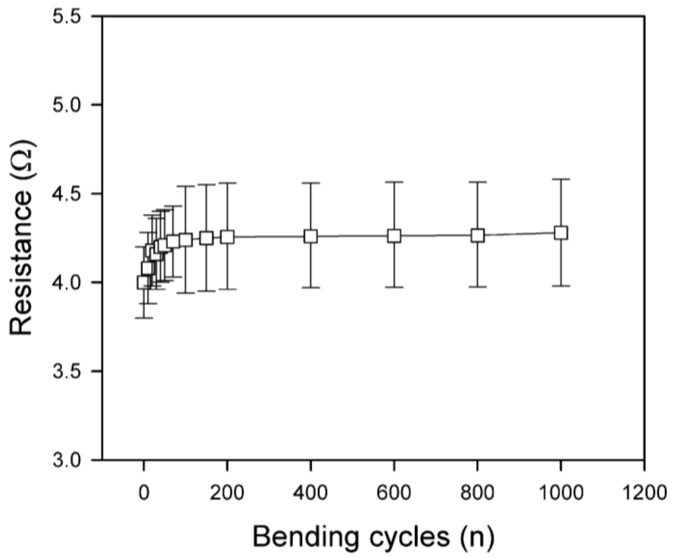

Figure S3. Electrical resistance change of the reduced graphene oxide/Ag Nanoparticle (rGO/AgNP) composite film on PET textile depending on the bending cycles.

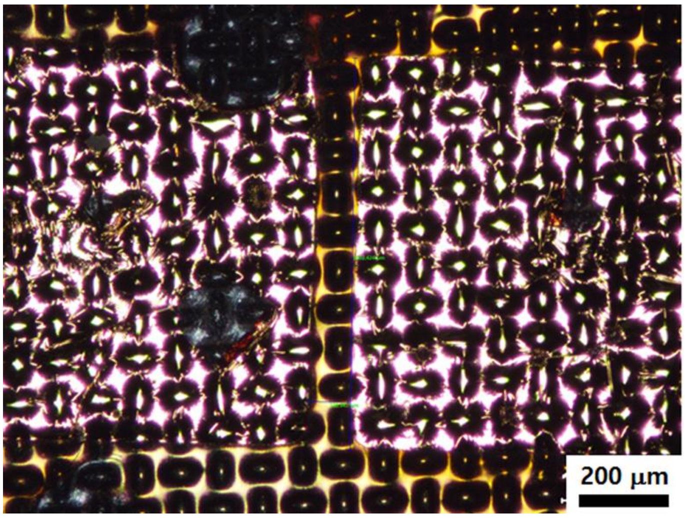

Figure S4. OM image of the textile-based transistor device after thermal annealing at 80 °C for 15 min.

© 2016 by the authors. Submitted for possible open access publication under the terms and conditions of the Creative Commons Attribution (CC-BY) license (http://creativecommons.org/licenses/by/4.0/).
